# Supplementary material for: A genome-wide screen identifies YAP/WBP2 interplay conferring growth advantage on human epidermal stem cells
Source: Nat Commun. 2017 Mar 23;8:14744. doi: 10.1038/ncomms14744 (PMC5376649; doi:10.1038/ncomms14744)
Supplement: Supplementary Information — Supplementary Figures, Supplementary Tables, Supplementary Methods and Supplementary References [file ncomms14744-s1.pdf]

Supplementary Figures

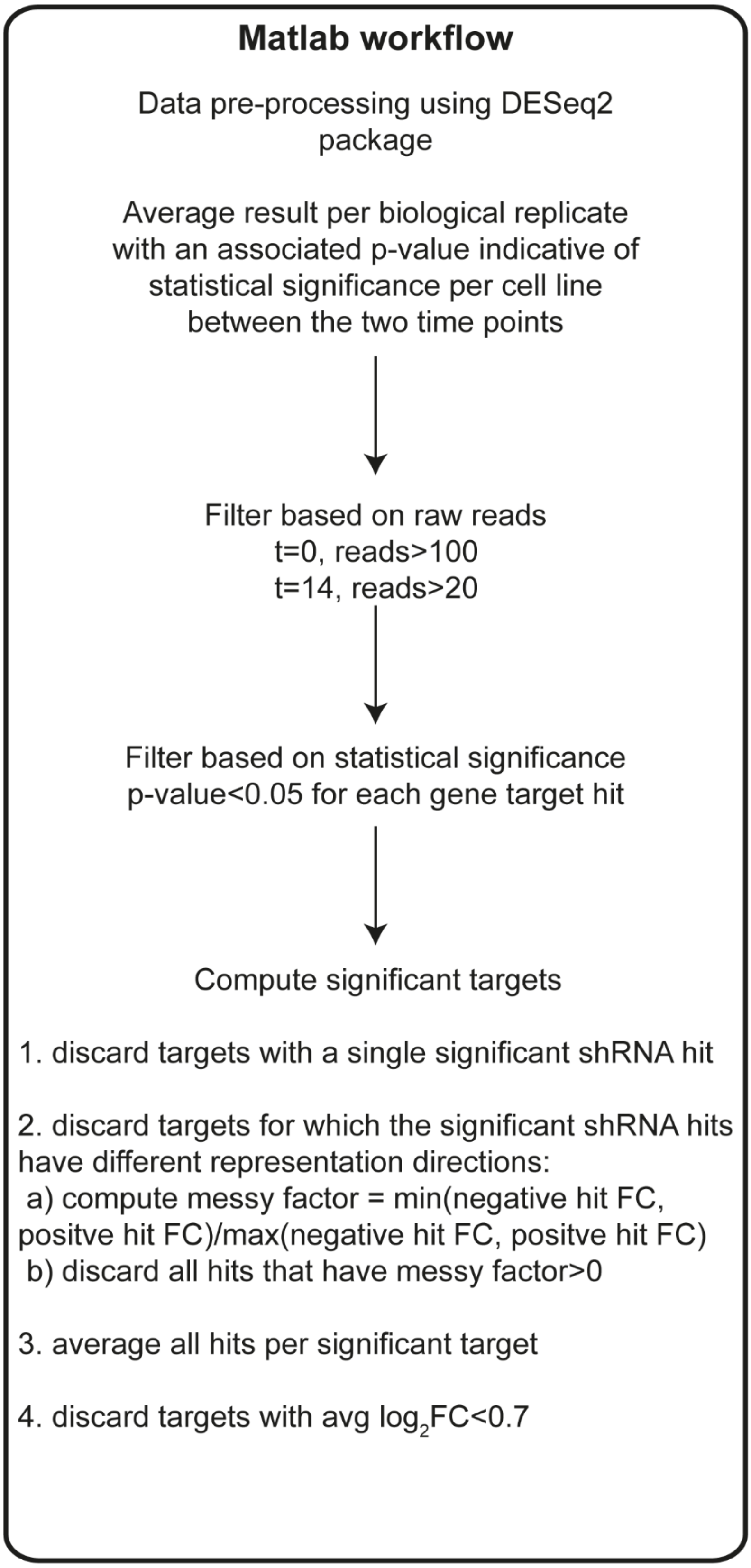

Supplementary Figure 1 Bioinformatics pipeline for gene target hit identification and ranking.

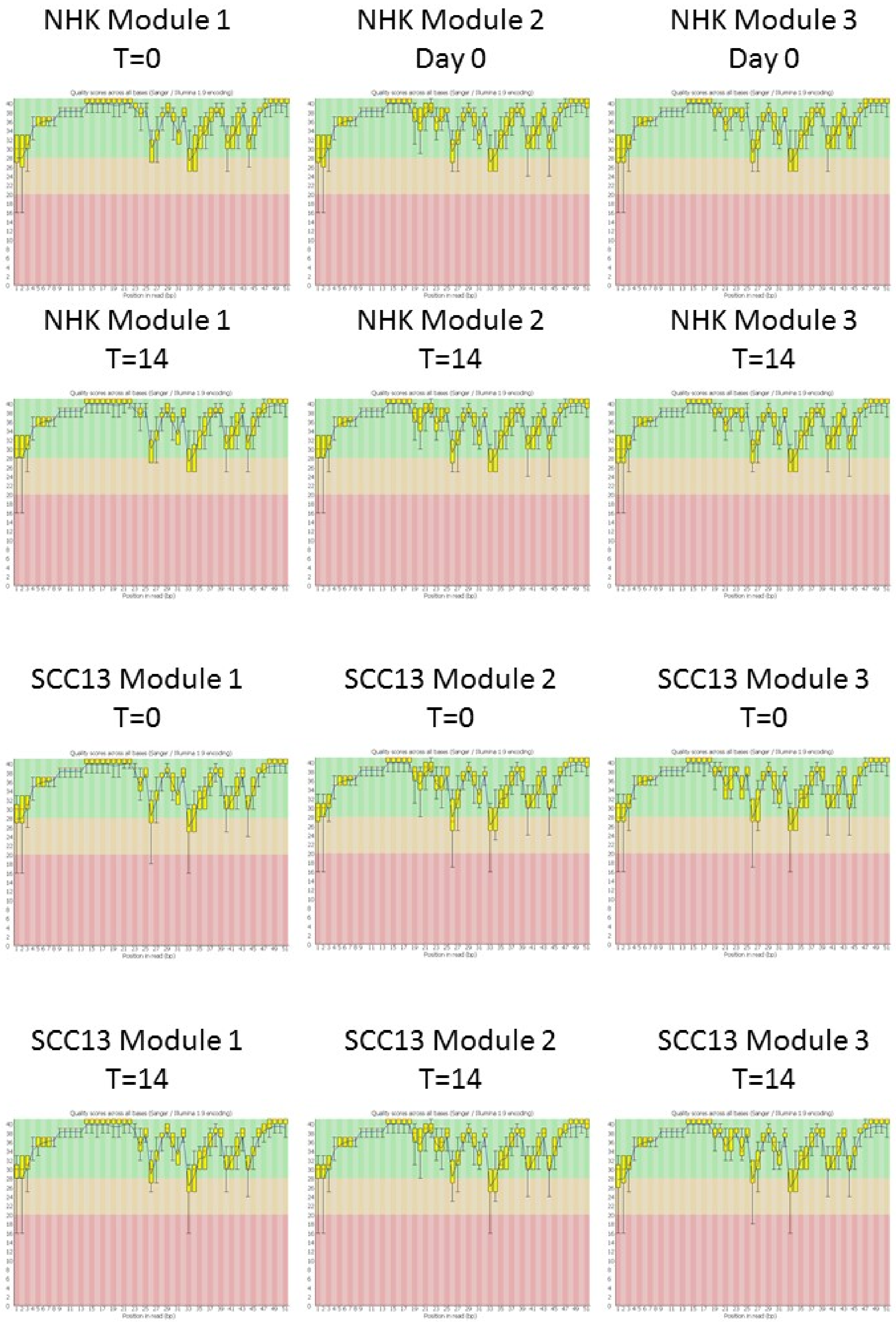

Supplementary Figure 2 Quality scores (x axes) across all bases (y axes) of the sequence reads of modules 1, 2 and 3 of the NHK and SCC13 screens (experiment 1, representative of

both experiments) at t=0 and t=14. The background of the graph divides the y axis into very good quality calls (green), calls of reasonable quality (orange), and calls of poor quality (red). Blue lines, average sequencing quality. Red lines, median values. Yellow boxes, inter-quartile ranges (25-75%). Upper and lower whiskers, 10th and 90th percentiles.

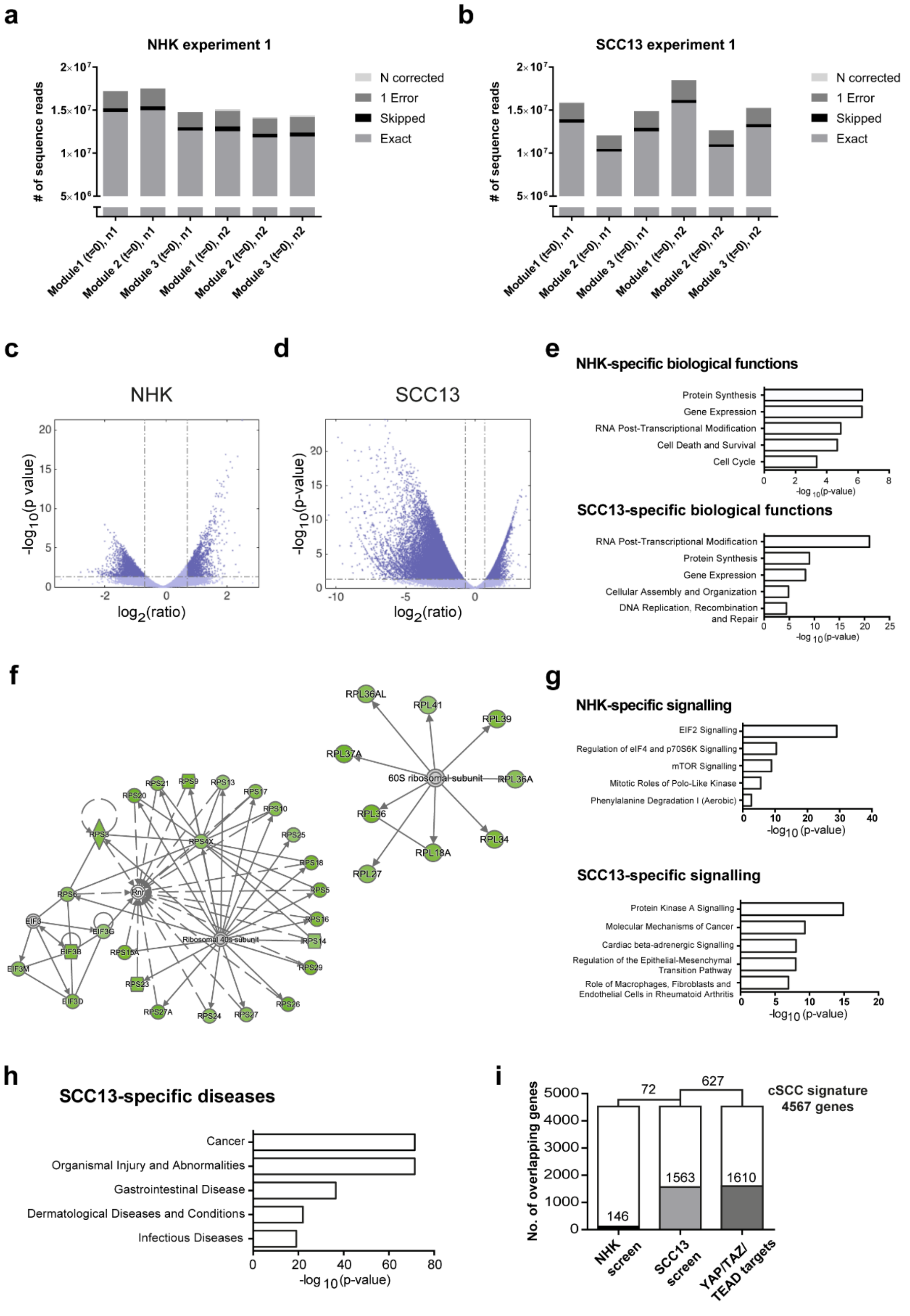

Supplementary Figure 3 Genome-wide pooled shRNA screens for growth regulators of NHKs and SCC13 cells. (**a, b**) Illumina sequencing performance. Total number of sequencing

reads across the three modules from the NHK and SCC13 screens (experiment 1, representative of both experiments) ranged between 1.2 and 1.85×10^8, with each module having over 82.5% of correctly identified shRNAs. (**c, d**) Significantly enriched or depleted shRNAs identified using DESeq2 on pooled datasets. Volcano plots show statistical significance ($-\log_{10}$(*p*-value)) versus relative shRNA abundance ($\log_2$-fold change) at t=14 days of 82,305 shRNAs in NHKs (**c**) and SCC13 cells (**d**). The vertical grey lines identify the $\log_2$-fold change cut-off (0.7) applied in the bioinformatics pipeline to identify gene target hits. The horizontal grey line identifies the cut-off applied in the bioinformatics pipeline for statistical significance ($p < 0.05$). (**e**) Putative growth regulators of NHKs and SCC13 cells are significantly enriched ($p < 0.05$) for gene function categories promoting cell proliferation and viability. (**f**) Components of the translational machinery complexes including 60S and 40S ribosomal subunits and eukaryotic initiation factor complex eIF3 were significantly overrepresented among genes identified as essential for NHK and SCC13 growth ($p < 0.05$). Colour schemes identify gene target hits whose respective shRNAs were significantly depleted during growth (green). (**g**) Putative growth regulators specific for either NHKs or SCC13 cells are enriched ($p < 0.05$) in distinct canonical signalling pathways. (**h**) Putative growth regulators specific for SCC13 cells are enriched ($p < 0.05$) in gene categories associated with cancer and organismal abnormalities. (**i**) A significant number of SCC13-specific gene target hits as well as bona-fide YAP/TAZ/TEAD target genes (Supplementary Data 8) match with a human cSCC gene signature (Supplementary Data 7).

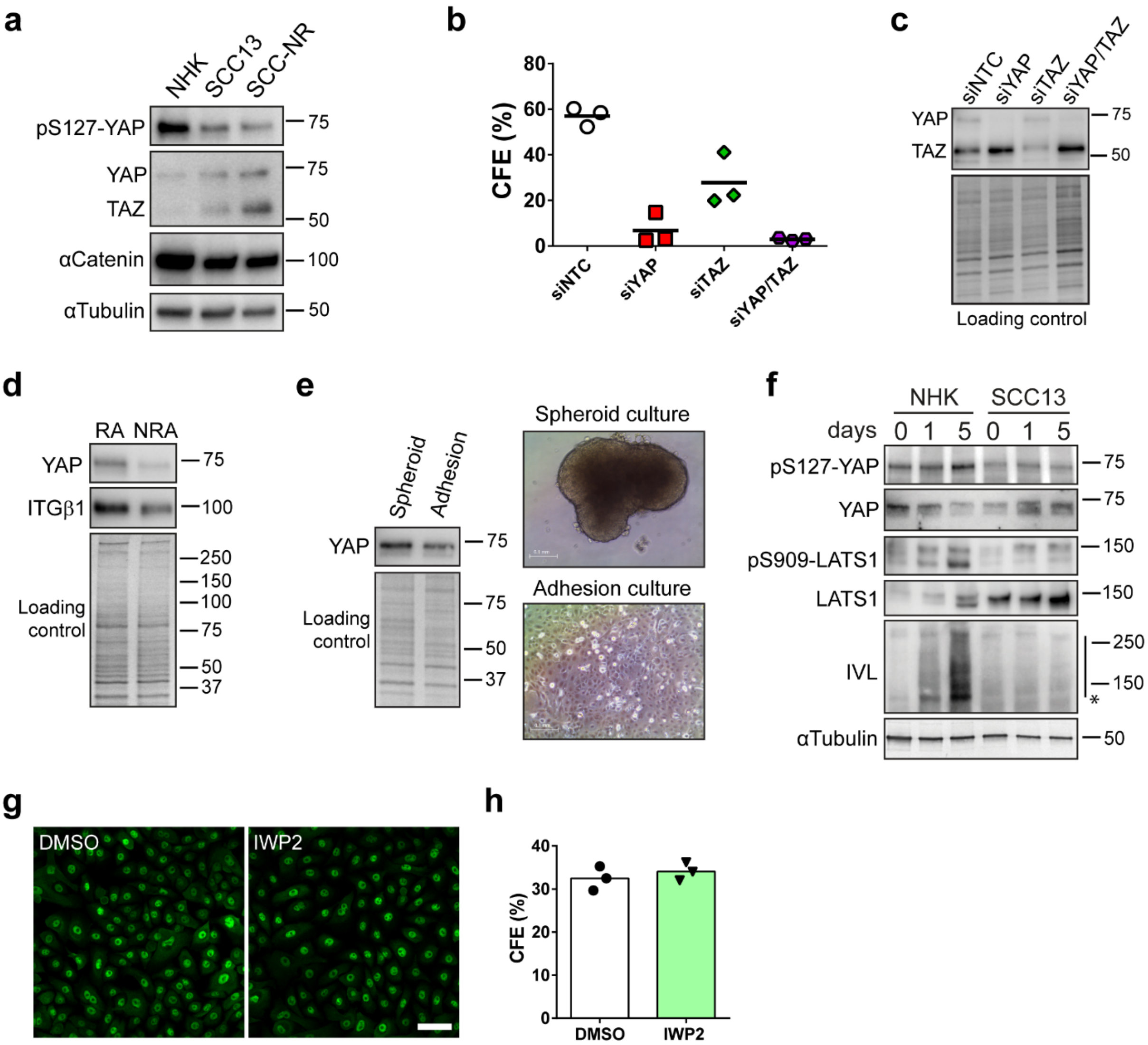

Supplementary Figure 4 Validation of YAP and TAZ as essential regulators of NHK and SCC13 cell growth. (**a**) Western blot analysis reveals reduced YAP S127 phosphorylation, increased YAP and TAZ expression, and decreased α-catenin expression in cSCC cells. NHKs, SCC13 cells and primary cSCC cells (SCC-NR) were grown as colonies on a fibroblast feeder layer, which was removed prior to cell lysis. Tubulin was used as loading control. (**b**) Clonal growth assay of SCC13 cells transfected with siRNA SMARTpools silencing YAP or TAZ. Note that YAP knock-down has a stronger effect on clonal growth than TAZ knock-down, despite compensatory upregulation of TAZ (**c**). Data shown are from three independent experiments performed with three technical replicates. Individual data points represent the average percentage of colonies formed per number of cells seeded (colony formation efficiency, CFE; bars represent the mean. (**c**) Western blot analysis of SCC13 cells transfected with siRNA SMARTpools silencing YAP or TAZ using antibodies detecting both YAP and

TAZ. Total protein content was monitored by enhanced tryptophan fluorescence imaging (Bio-Rad) of PVDF membranes (loading control). (**d, e**) Western blot analysis of cell fractions enriched for cSCC-associated SCs using antibodies against YAP and ITGβ1. Equal protein loading was confirmed by enhanced tryptophan fluorescence imaging (Bio-Rad) of PVDF membranes (loading control). (**d**) Rapidly adhering (RA) SCs were separated from non-rapidly adhering (NRA) SCC13 cells based on their faster adhesion to collagen type IV. (**e**) cSCC-associated SCs were enriched for by growing SCC13 cells as spheroids, and compared to cells grown as adhesion cultures. (**f**) NHKs and SCC13 cells were grown to confluence in calcium-reduced/serum-free medium (KSFM, day 0) in the absence of a feeder layer, switched to calcium/serum-containing FAD medium to induce cell-cell contact and stratification of terminally differentiating cells, and cultured over a time course of 5 days (days 1 and 5). Cell lysates were subjected to western blot analysis using antibodies against pS127-YAP, total YAP, LATS1, auto-phosphorylated (activated) pS909-LATS1, and involucrin (IVL). The asterisk indicates the monomeric involucrin band (~140 kD); transglutaminase-crosslinked involucrin multimers generated upon terminal differentiation are indicated with a vertical bar. Tubulin was used as loading control. (**g**) Representative YAP immunostainings (maximum intensity projections) of NHKs cultured for 24 hours in the presence of the WNT-antagonist IWP2 (4 µM) or vehicle (DMSO) alone. Scale bar, 50 µm. (**h**) Clonal growth assays of NHKs in the presence of IWP2 (4 µM) or DMSO alone. Data shown are from three independent experiments performed with three technical replicates. Individual data points represent the average percentage of colonies formed per number of cells seeded (colony formation efficiency, CFE), bars represent the mean.

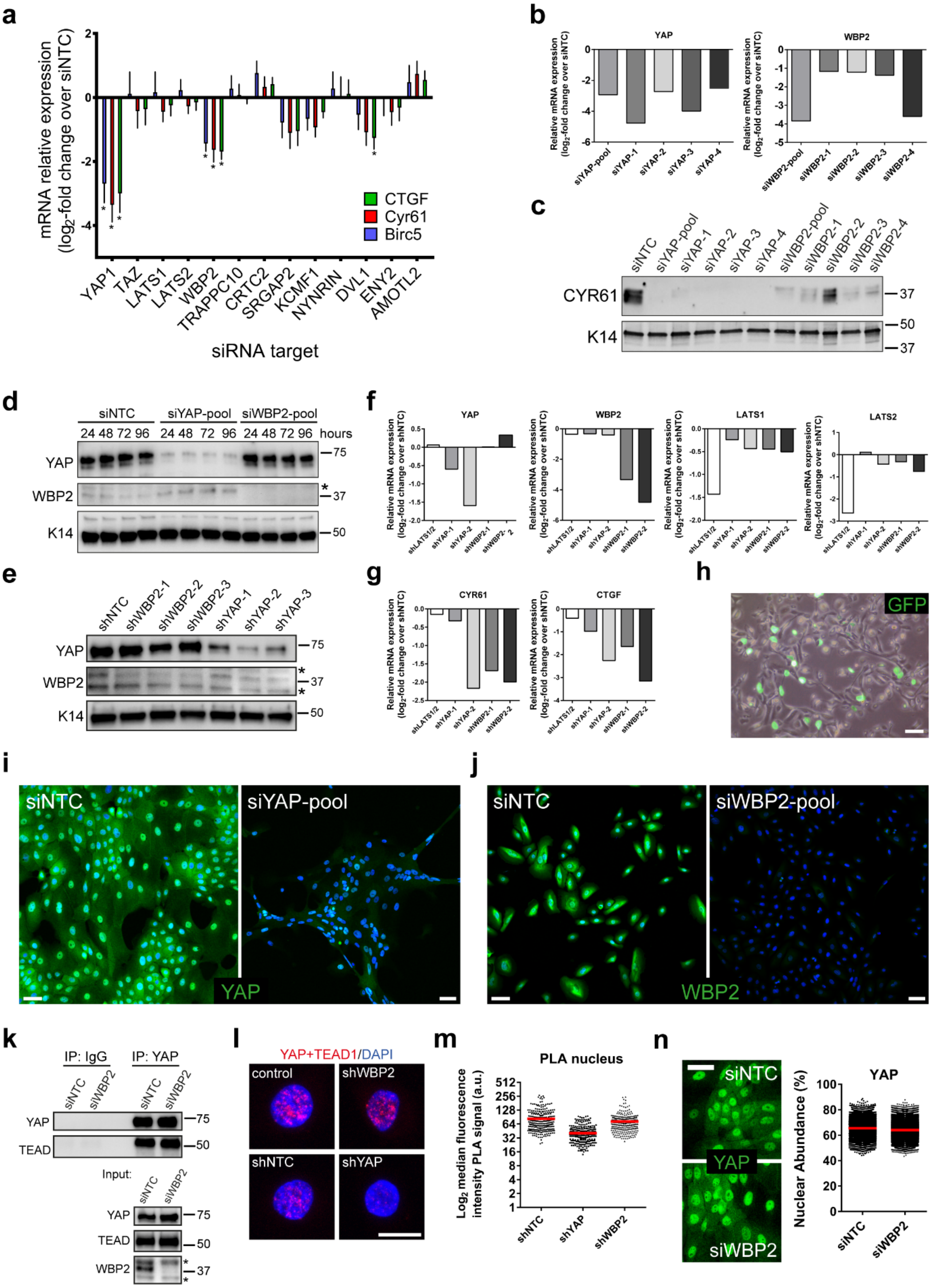

Supplementary Figure 5 YAP regulator siRNA screen deconvolution and antibody specificity controls. (**a**) Q-RT PCR analysis of mRNA expression levels of YAP/TAZ/TEAD target genes in NHKs transfected with siRNA SMARTpools targeting selected candidate YAP regulators

and cultured for 48 hours. Data are from 5 independent experiments performed with two biological replicates (independent siRNA transfections). Bars represent mean±S.E.M. of the average $\log_2$-fold change in mRNA abundance (normalized to 18sRNA) compared to siNTC in each experiment. * $p < 0.05$, two-way ANOVA with Dunnett's multiple comparison test. (**b–c**) siRNA screen deconvolution. NHKs were transfected with the individual siRNAs comprising the SMARTpools used to silence YAP and WBP2 in the siRNA screen and a non-targeting (NTC) control siRNA, and cultured for up to 96 hours. (**b**) Q-RT PCR analysis of YAP and WBP2 mRNA expression 48 hours after siRNA transfection. Data shown are from one representative experiment performed with four technical replicates. Bars represent the mean $\log_2$-fold change in mRNA abundance (normalized to 18sRNA) compared to siNTC. (**c**) Cell lysates were subjected to western blot analysis using antibodies against the YAP/TAZ/TEAD target CYR61. Keratin (K)14 was used as loading control. (**d**) NHK cell lysates prepared at different times after transfection with siRNA SMARTpools were subjected to western blot analysis using antibodies against YAP and WBP2. Keratin (K)14 was used as loading control. Asterisk indicates unspecific bands observed with anti-WBP2 antibodies. (**e–g**) NHKs stably expressing different YAP- or WBP2-specific shRNAs, LATS1 and 2-specific shRNAs, or a non-targeting control (NTC) shRNA were subjected to western blot (**e**) or q-RT PCR (**f, g**) analysis. (**e**) Western blot analysis using the indicated antibodies. Keratin (K)14 was used as loading control. Asterisks indicate unspecific bands observed with anti-WBP2 antibodies. (**f, g**) Quantitative real-time PCR analysis of the indicated shRNA-targeted genes (**f**) or YAP/TAZ/TEAD target genes (**g**) in NHKs 7 days after lentiviral infection. Data shown are from one representative experiment performed with four technical replicates. Bars represent the mean $\log_2$-fold change in mRNA abundance (normalized to 18sRNA) compared to shNTC. (**h**) Expression of GFP-IRES-shYAP construct 24 hours after lentiviral infection. Scale bar, 20 µm. (**i, j**) Validation of YAP and WBP2 antibodies for immunostainings and in situ proximity ligation assays. NHKs were transfected with siRNA SMARTpools to silence YAP or WBP2, cultured for 48 hours, and immunolabelled using YAP- or WBP2-specific antibodies. Representative images (maximum intensity projections) are shown. Scale bars, 20 µm. (**k**) YAP co-immunoprecipitation with TEADs at endogenous protein levels in NHKs is not affected by siRNA-mediated knock-down of WBP2. Immunoprecipitates and input lysates were subjected to western blot analysis using using antibodies against the indicated proteins. Two independent immunoprecipitations were performed with similar results. (**l**) Representative images (maximum intensity projections) of in situ PLA detection of endogenous nuclear YAP/TEAD1 interactions in NHKs expressing the indicated shRNAs. Scale bars, 25 µm. (**m**)

High content imaging-based semi-automated quantification of nuclear YAP/TEAD1 PLA signals (individual data points) in NHKs expressing the indicated shRNAs. One representative experiment with 250 cells analysed per condition is shown in each case. Red lines represent the mean. (**n**) Representative YAP immunostainings (maximum intensity projections) and high content imaging-based semi-automated quantification of nuclear YAP abundance (individual data points and their means, red lines) in NHKs transfected with a siRNA SMARTpool targeting WBP2, or a non-targeting (NTC) siRNA. One representative experiment with 10,000 cells analysed per condition is shown. Scale bar, 20 µm.

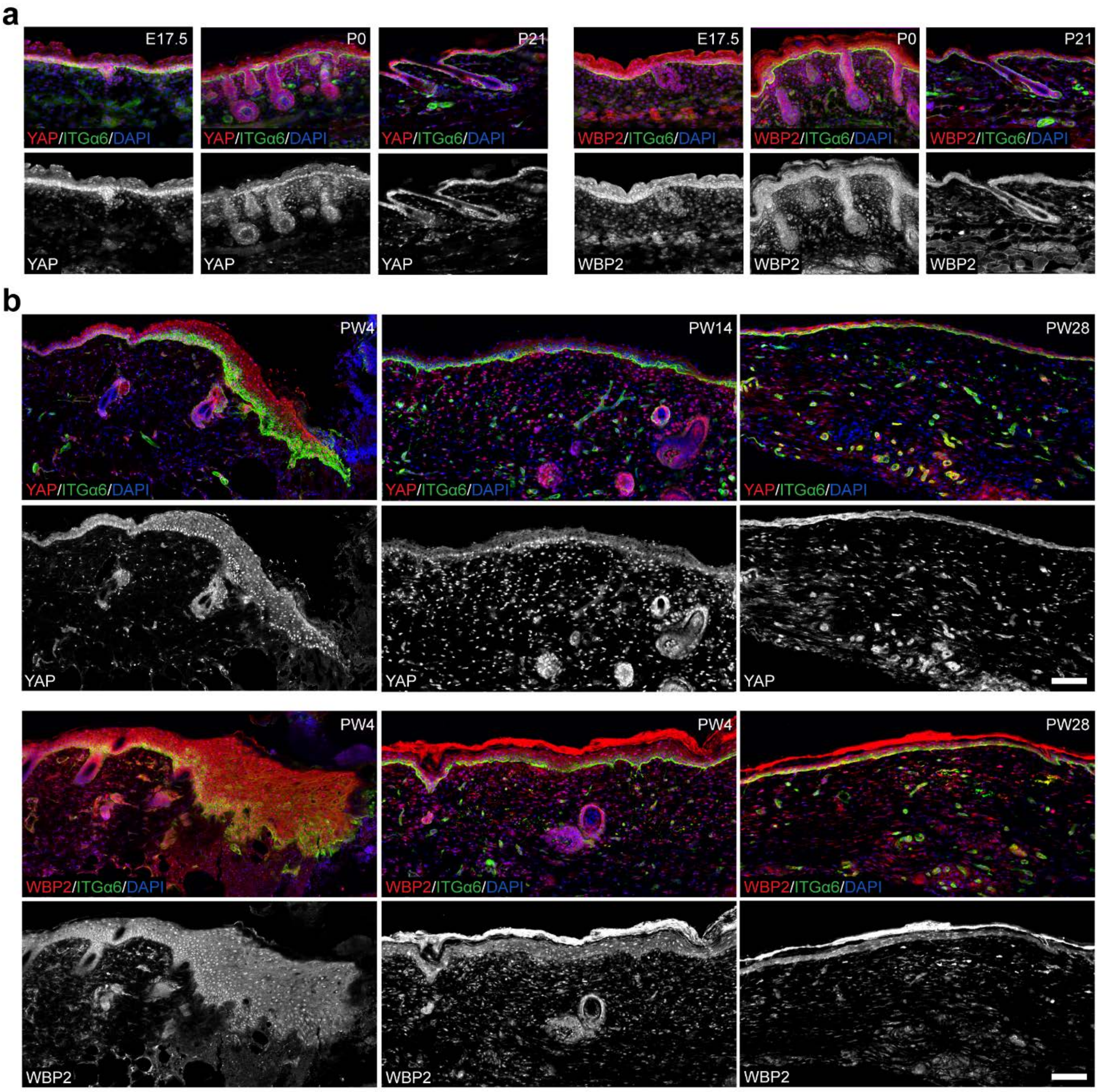

Supplementary Figure 6 Expression of YAP/WBP2 in mouse skin development, homeostasis and wound healing. (**a, b**) Representative images of mouse back skin samples at the indicated age (**a**) or at indicated days after full thickness wounding (**b**). Sections were immunolabelled with the indicated antibodies and counterstained with DAPI to reveal nuclei. Scale bars, 100 µm. E, embryonic day; P, postnatal day; PW, post-wounding day.

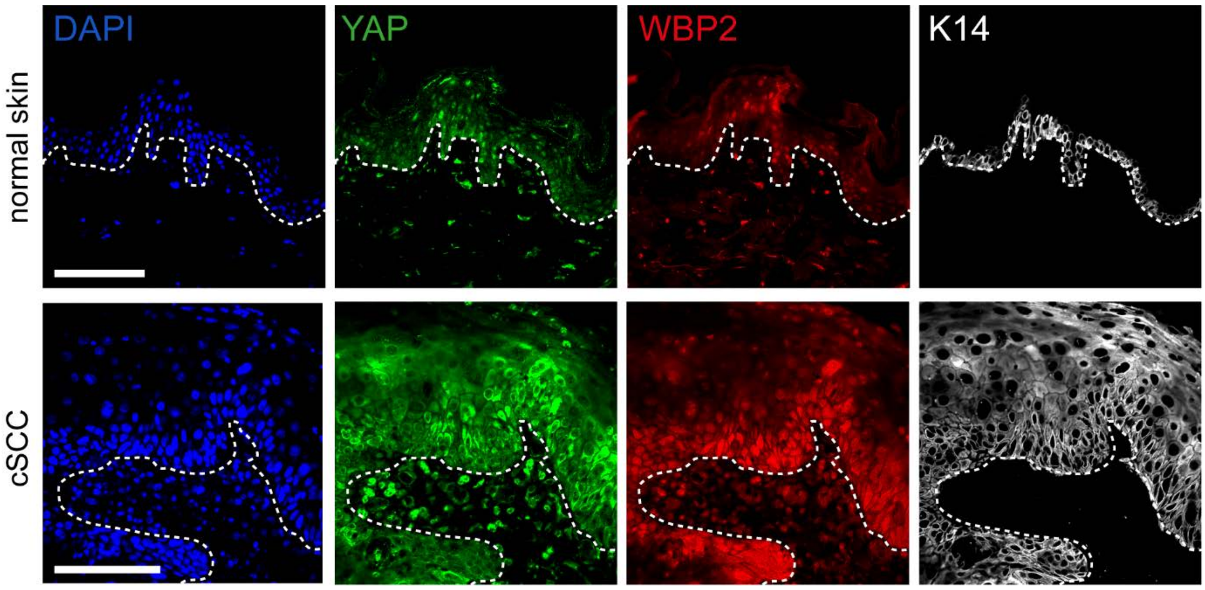

Supplementary Figure 7 Increased expression of YAP and WBP2 in human cutaneous SCC. Representative images of normal adult human skin and cutaneous SCC (cSCC) sections immunolabelled with antibodies against YAP and WBP2 and counterstained with DAPI to reveal nuclei. White dotted lines demarcate dermal–epidermal boundary in normal skin and tumour-stroma interface in tumour samples. Scale bars, 100 µm.

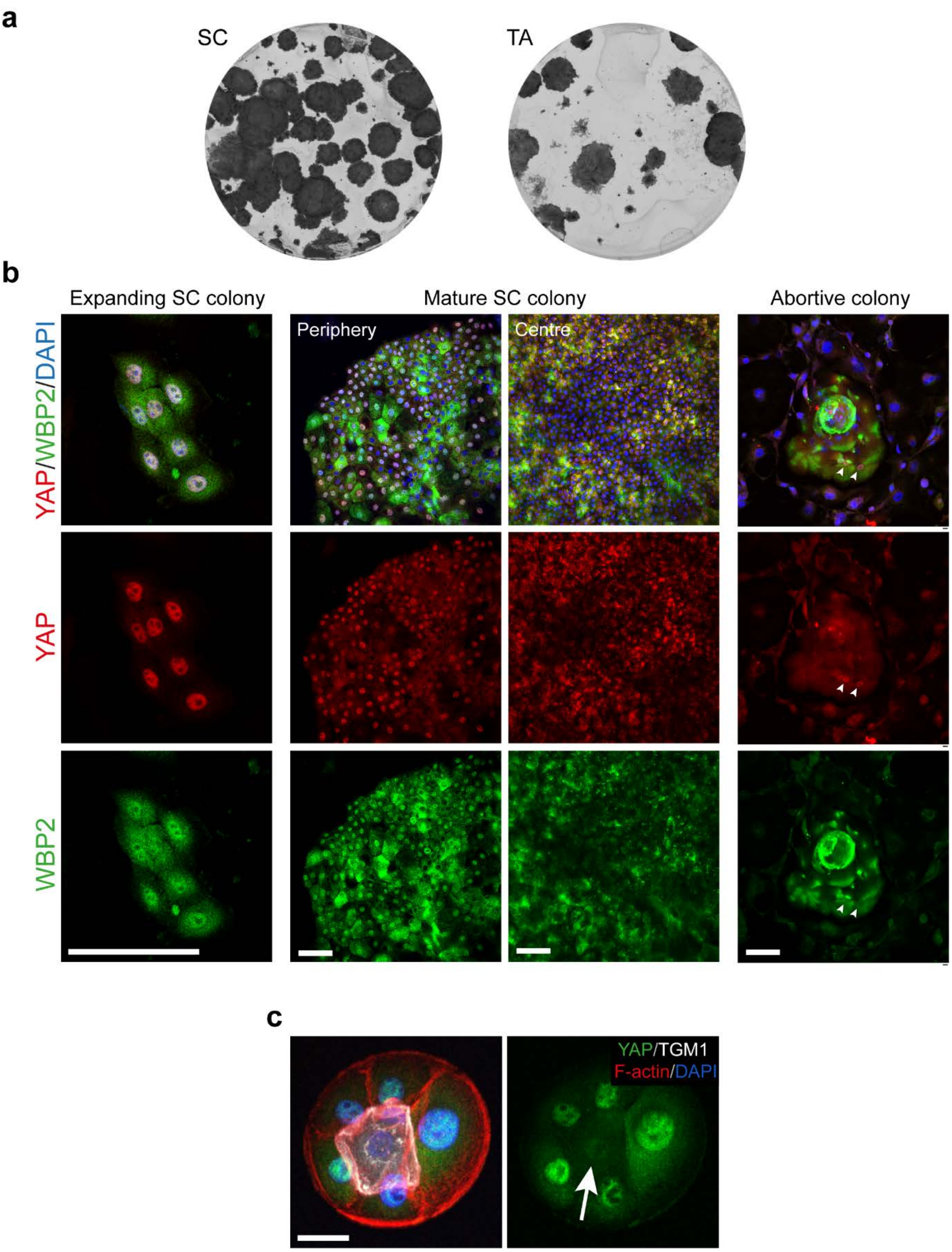

Supplementary Figure 8 Subcellular localization of YAP and WBP2 differs in NHK colonies with different proliferative potentials. (**a**) Representative images of Rhodanile Blue-stained colonies formed after seeding NHK fractions enriched for SCs or transit amplifying (TA) cells

onto a fibroblast feeder layer, and culturing them for 14 days. Similar dishes were used for immunostainings in Fig. 3g. (**b**) Split channel images (maximum intensity projections) corresponding to Fig. 3g. Arrowheads denote terminally differentiated in abortive colonies cells displaying nuclear YAP but not WBP2. Scale bars, 100 µm. (**c**) Representative image (volume view) of a micro-epidermis formed by NHKs on 80 µm circular adhesive micro-islands. Cells were immunolabeled with indicated antibodies and counterstained with Phalloidin and DAPI to reveal F-actin and nuclei, respectively. The arrow indicates loss of nuclear YAP in a terminally differentiating (TGM1-positive) cell which is in the process of delamination from the substrate and stratification on top of the other basal cells. Scale bar, 25 µm.

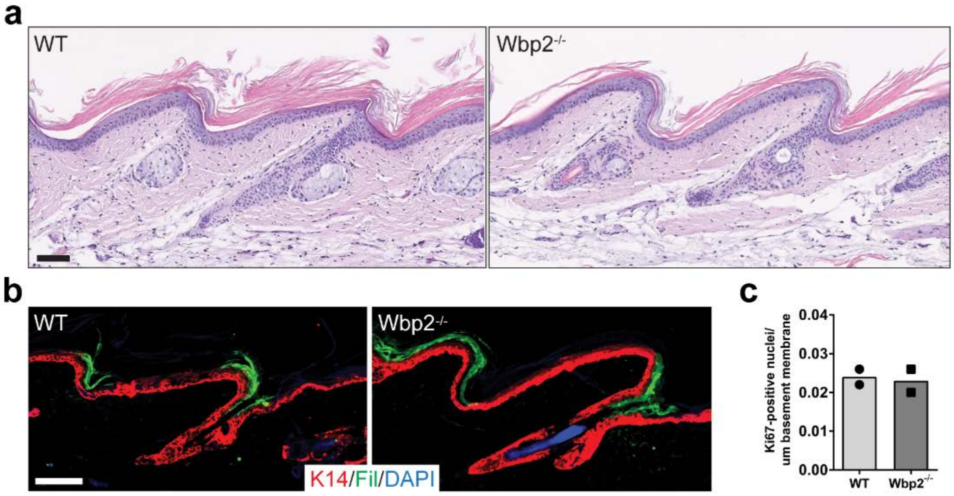

Supplementary Figure 9 Phenotypic analysis of wild-type (WT) and WBP2-null (Wbp2$^{-/-}$) mouse littermates. (**a, b**) Representative images of tail skin samples of adult mice. Sections were either H&E-stained (**a**) or immunolabelled with the indicated antibodies (Fil, filaggrin) and counterstained with DAPI to reveal nuclei (**b**). Scale bars: **a,** 100 µm; **b,** 50 µm. (**c**) Quantification of basal cells positive for Ki67 in back skin epidermis. Individual data points represent the average number of Ki67-positive basal epidermal cells per µm of basement membrane, quantified from four immunolabelled sections representing different back skin areas. Bars represent the mean.

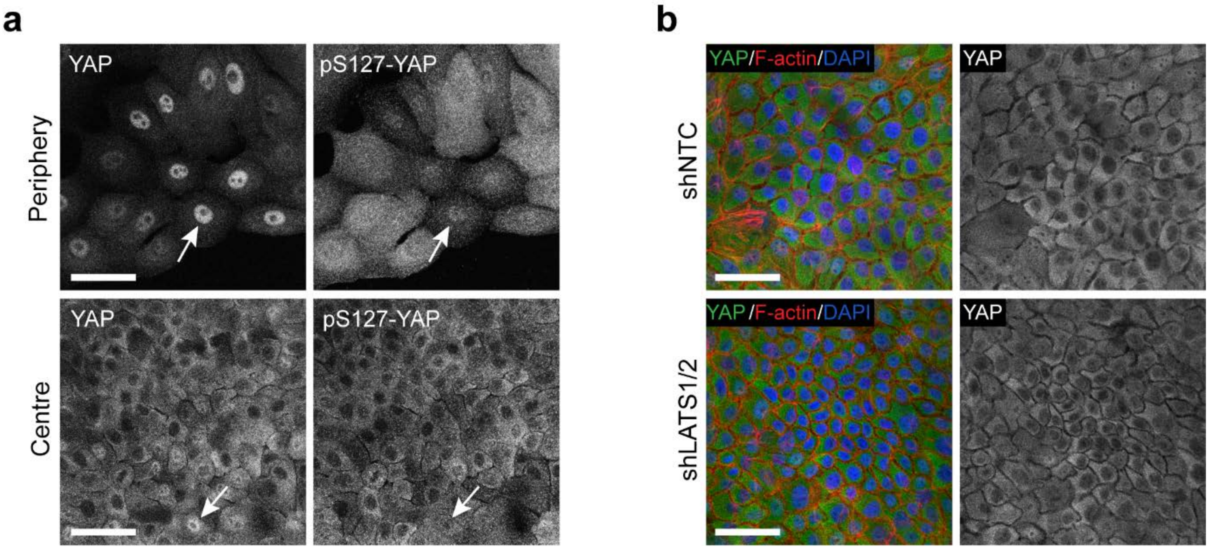

Supplementary Figure 10 LATS1/2 mediated phosphorylation of YAP at S127 does not affect YAP localisation (**a, b**) Representative images (maximum intensity projections covering the basal cell layer) of mature SC colonies immunolabelled with the indicated antibodies and counterstained with DAPI or phalloidin to reveal nuclei and F-actin, respectively. Cells in (**b**) were expressing either a non-targeting control shRNA (shNTC) or LATS1/2-silencing shRNAs. Arrows in (**a**) point at cells with nuclear localization of both total and S127-phosphorylated YAP. Scale bar, 100 µm.

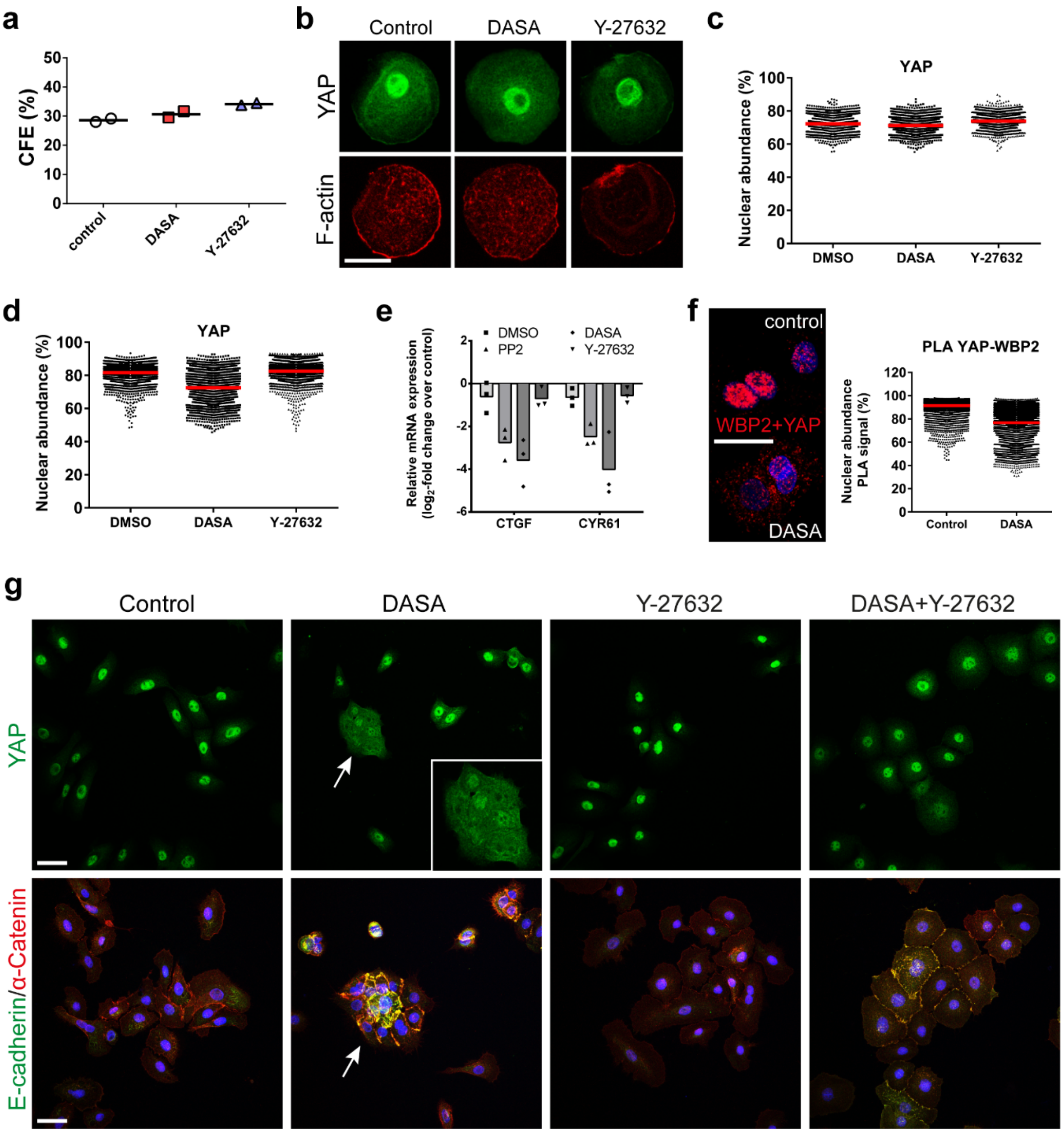

Supplementary Figure 11 SFKs and ROCK regulate YAP/WBP2 via modulation of adherens junctions stability. (**a**) Colony formation efficiency of NHKs treated as in Fig. 7b–g. Data shown are from two independent experiments performed with three technical replicates. Individual data points represent the average percentage of colonies formed per number of cells seeded, lines represent the mean. (**b–g**) NHKs were either captured on arrays of micro-patterned 20- and/or 50 µm circular adhesive islands (**b, c**) on non-patterned substrates (**d–g**) and cultured in the absence or presence of Dasatinib (DASA, 2 µM) or Y-27632 (10 µM) for 24 hours. Note that we used 2 µM Dasatinib instead of 4 µM when NHKs were cultured in KSFM medium because we found the cells to be more sensitive to the drug than when cultured in serum-containing medium in the presence of feeder cells. (**b**) Representative images

(maximum intensity projections) of cells captured on 50 µm adhesive islands show YAP immunostainings and F-actin labelling (phalloidin). Scale bar, 25 µm. (**c, d**) High content imaging-based semi-automated quantification of nuclear YAP abundance (individual data points). One representative experiment with 1500 (**c**) or 3000 (**d**) cells analysed per condition is shown. Red lines represent the mean. (**e**) Q-RT PCR analysis of YAP/TAZ/TEAD target gene expression. Data shown are from three independent experiments performed with two biological replicates (independent drug treatments). Individual data points represent the average log_2-fold change in mRNA abundance (normalized to 18sRNA) compared to control in each experiment. Bars represent the mean. (**f**) In situ proximity ligation assay (PLA) detection of endogenous nuclear YAP/WBP2 interactions in NHKs (individual data points and their means, red lines). One representative experiment with 7000 cells analysed per condition is shown. Scale bar, 50 µm. (**g**) Representative YAP and E-cadherin/α-catenin immunostainings (maximum intensity projections) of NHKs captured on non-patterned substrates and cultured for 24 hours in the presence of Dasatinib (2 µM) or Y-27632 (10 µM) alone, a combination of Dasatinib and Y-27632, or vehicle (DMSO) alone. Cells were counterstained with DAPI to reveal nuclei. Arrows and inset, micro-colonies formed upon Dasatinib treatment. Scale bars, 50 µm.

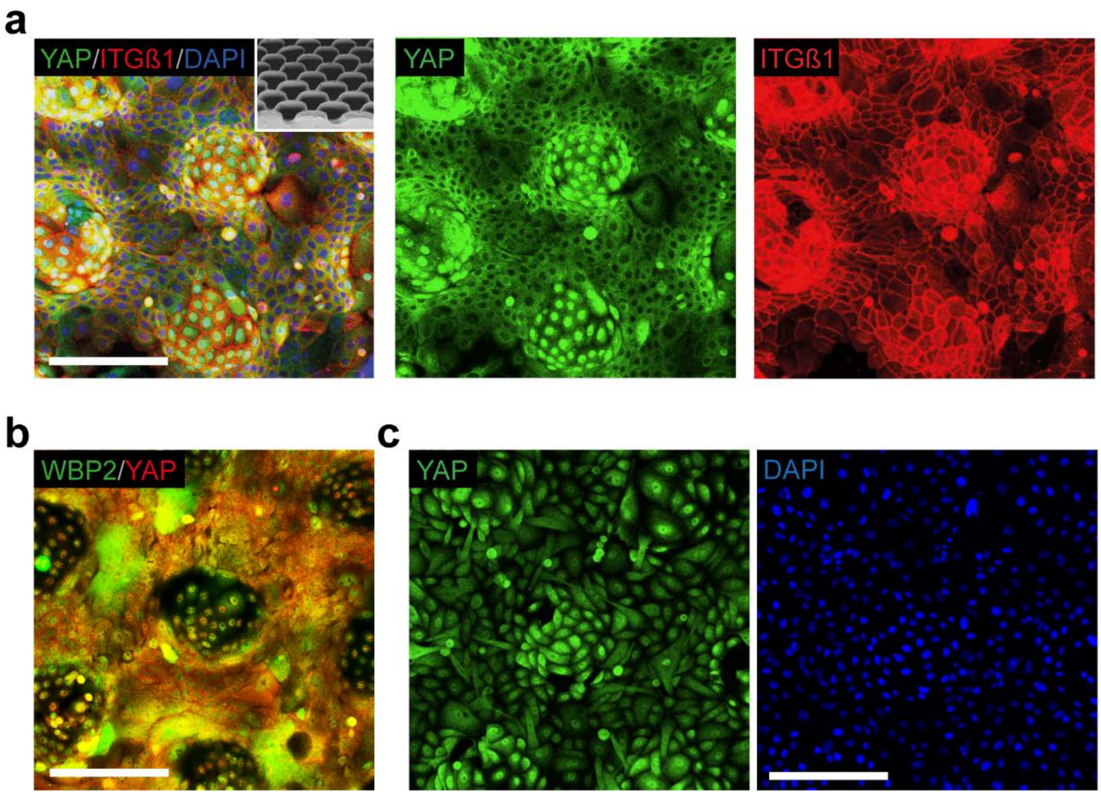

Supplementarty Figure 12 Cell-cell adhesion-dependent patterning of nuclear YAP within epidermal sheets. (**a–c**) Representative images (maximum intensity projections) of NHKs grown on PDMS substrates with patterned topographical features (insert in (**a**), scanning electron micrograph), and immunolabelled with the indicated antibodies and counterstained for DAPI to reveal nuclei. Cells were either grown in high Ca^{++} FAD medium (**a, b**), or in low Ca^{++} FAD medium (**c**). Scale bar, 200 µm.

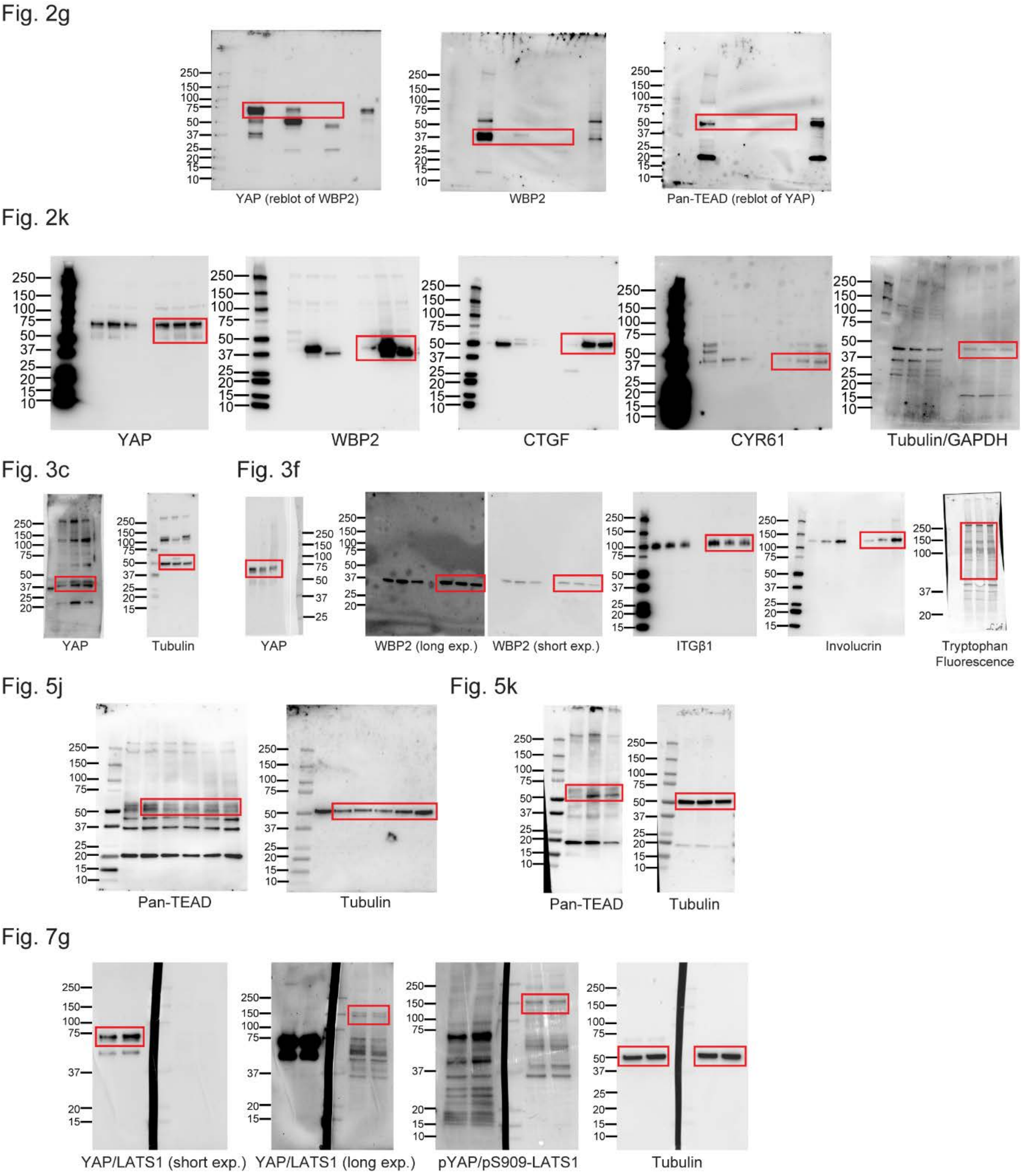

Supplementary Figure 13 Uncropped western blots.

Fig. S4a

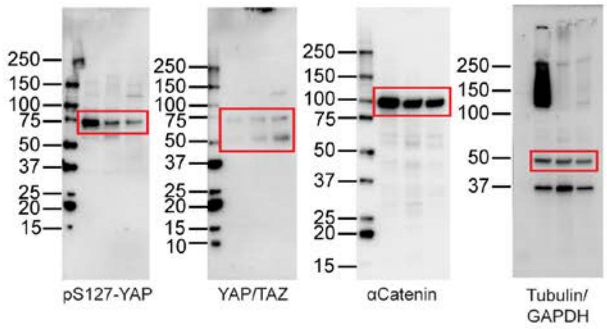

Fig. S4c

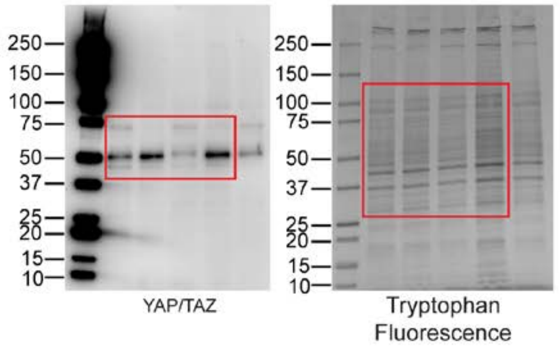

Fig. S4d

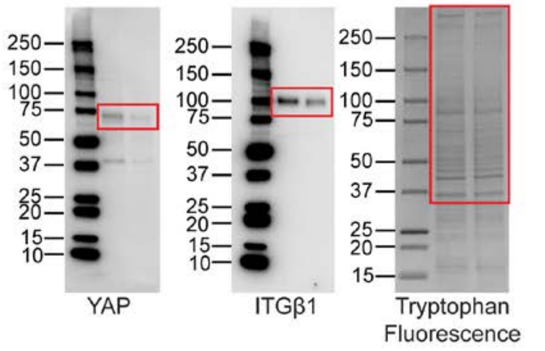

Fig. 4e

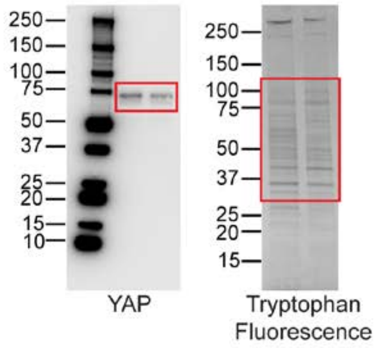

Fig. S4f

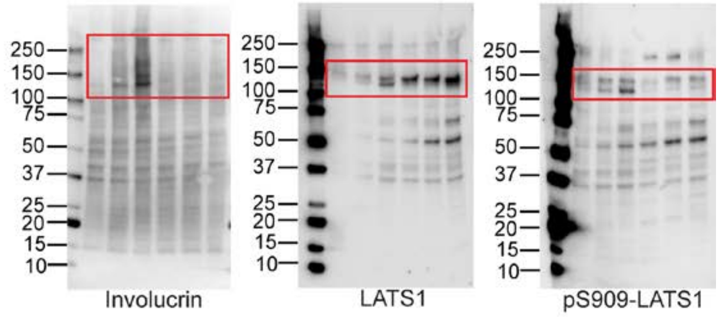

Fig. S4f (continued)

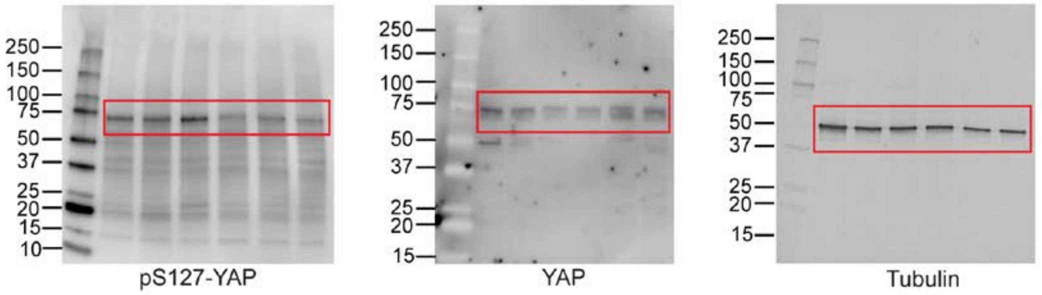

Supplementary Figure 13 (continued) Uncropped western blots.

Fig. S5c

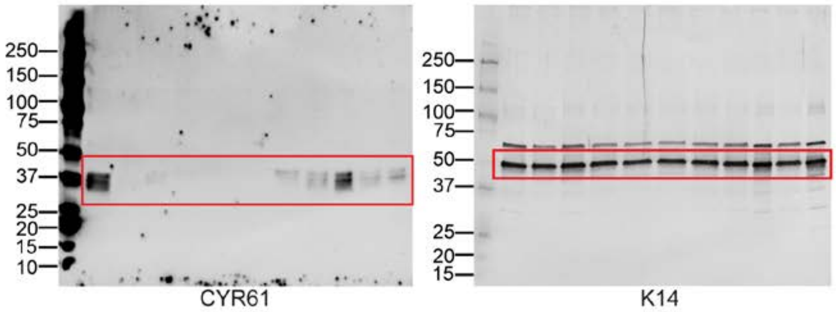

Fig. S5d

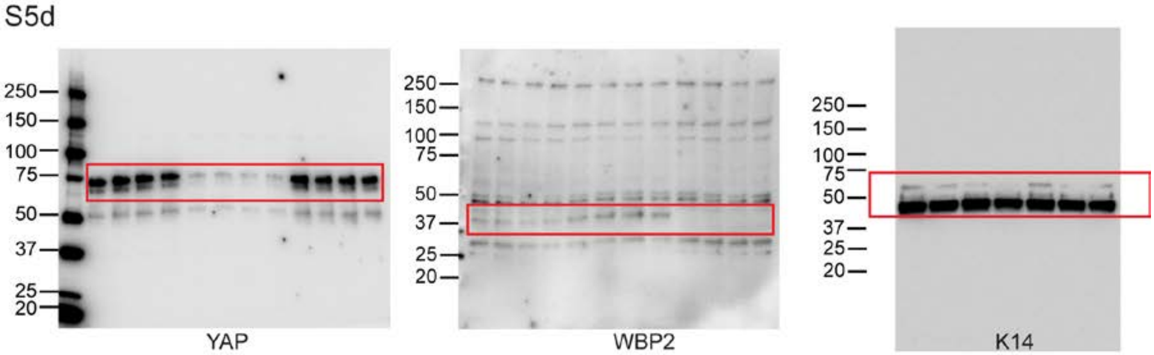

Fig. S5e

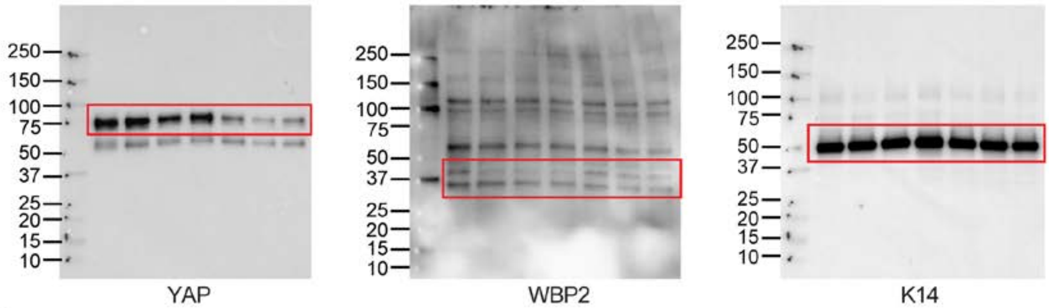

Fig. S5h

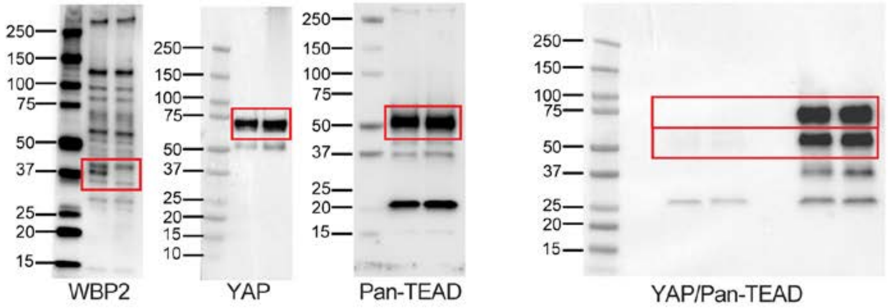

Supplementary Figure 13 (continued) Uncropped western blots.

Supplementary Tables

Supplementary Table 1 List of MISSION shRNA lentiviral transduction particles

Product Number	TRC Number	Gene target	Clone ID	Sequence
SHCLNV-NM_006106	TRCN0000107267	YAP1	NM_006106.2-1373s1c1	CCGGCAGGTGATACTATCAACCAAACTCGAGTTTGGTTGATAGTATCACCTGTTTTTG
SHCLNV-NM_006106	TRCN0000300281	YAP1	NM_006106.3-1354s21c1	CCGGGACCAATAGCTCAGATCCTTTCTCGAGAAAGGATCTGAGCTATTGGTCTTTTTG
SHCLNV-NM_006106	TRCN0000300282	YAP1	NM_006106.3-2049s21c1	CCGGCCCAGTTAAATGTTCACCAATCTCGAGATTGGTGAACATTTAACTGGGTTTTTG
SHCLNV-NM_012478	TRCN0000331067	WBP2	NM_012478.3-204s21c1	CCGGCGTGGAACTCACATTCAATGACTCGAGTCATTGAATGTGAGTTCCACGTTTTTG
SHCLNV-NM_012478	TRCN0000353780	WBP2	NM_012478.3-153s21c1	CCGGCGGCGGAGTGATCGTCAATAACTCGAGTTATTGACGATCACTCCGCCGTTTTTG
SHCLNV-NM_012478	TRCN0000331002	WBP2	NM_012478.3-1800s21c1	CCGGCCATGAAGTGAGGAGATGGTTCTCGAGAACCATCTCCTCACTTCATGGTTTTTG
SHCLNV-NM_004690	TRCN0000001777	LATS1	NM_004690.x-3151s1c1	CCGGCACGGCAAGATAGCATGGATTCTCGAGAATCCATGCTATCTTGCCGTGTTTTT
SHCLNV-NM_014572	TRCN0000000880	LATS2	NM_014572.x-3750s1c1	CCGGCCGTCGATTACTTCACTTGAACTCGAGTTCAAGTGAAGTAATCGACGGTTTTT

Supplementary Table 2 List of qPCR primers

**Gene**	**Forward primer**	**Reverse primer**
18sRNA	GCAATTATTCCCCATGAACG	GGCCTCACTAAACCATCCAA
CYR61	TGCAGAGCTCAGTCAGAGGGCA	ACCAGCCGAGGGTTGGGACA
CTGF	GCGTGTGCACCGCCAAAGATG	GTCCATGCTGCACAGGGGCAT
BIRC5	GCCAGATGACGACCCCATGCAA	CACGGCGCACTTTCTCCGCA
YAP1	GGCGCTCTTCAACGCCGTCA	GTACTGGCCTGTCGGGAGTGGG
WBP2	GCCCCCACCTGAGTTCTATC	TGGGTTGTAATAGGCGCTGG
TGM1	GTTGCCCTTTGACCCCCGCA	CCCCGTGGTCAAACTGGCCG
IVL	GCCTCAGCCTTACTGTGAGT	TGTTTCATTTGCTCCTGATGG
LATS1	ATTAGAGCGGAGAGCTGCAC	TGTGTGTATCCTGTTCGTAGCA
LATS2	CTAACTGTCGGTGTGGGGAC	TGAGTGTACCCTTTGCGGAG

Supplementary Table 3 List of On-TARGET plus SMARTpool siRNAs

Pool Catalog Number	Duplex Catalog Number	Gene Symbol	GENE ID	Gene Accession	Sequence
L-013232-01	J-013232-09	AMOTL2	51421	NM_016201	UCUUCUAGUGAGCGACAAA
L-013232-01	J-013232-10	AMOTL2	51421	NM_016201	CCAAAGCCCACUCGCAGUA
L-013232-01	J-013232-11	AMOTL2	51421	NM_016201	CAUAAAGUUUUGUCCGAAA
L-013232-01	J-013232-12	AMOTL2	51421	NM_016201	UCAGAACAACUGCGAGAGA
L-004632-00	J-004632-05	LATS1	9113	NM_004690	GGUGAAGUCUGUCUAGCAA
L-004632-00	J-004632-06	LATS1	9113	NM_004690	UAGCAUGGAUUUCAGUAAU
L-004632-00	J-004632-07	LATS1	9113	NM_004690	GGUAGUUCGUCUAUAUUAU
L-004632-00	J-004632-08	LATS1	9113	NM_004690	GAAUGGUACUGGACAAACU
L-003865-00	J-003865-09	LATS2	26524	NM_014572	GCACGCAUUUUACGAAUUC
L-003865-00	J-003865-10	LATS2	26524	NM_014572	ACACUCACCUCGCCCAAUA
L-003865-00	J-003865-11	LATS2	26524	NM_014572	AAUCAGAUAUUCCUUGUUG
L-003865-00	J-003865-12	LATS2	26524	NM_014572	GAAGUGAACCGGCAAAUGC
L-009608-00	J-009608-05	TAZ	6901	NM_181313	GGGACUGGGUGCAUAUCUU
L-009608-00	J-009608-06	TAZ	6901	NM_181313	GGACCAAGUACAUGAACCA
L-009608-00	J-009608-07	TAZ	6901	NM_181313	GCGCCUGAUUGCUGAGUGU
L-009608-00	J-009608-08	TAZ	6901	NM_181313	UGAAUGACGUCCUUCCUAA
L-018947-00	J-018947-05	CRTC2	200186	NM_181715	GGCUAAACAUGCUGAGUGA
L-018947-00	J-018947-06	CRTC2	200186	NM_181715	GCAAAUUUAGUGAGAAGAU
L-018947-00	J-018947-07	CRTC2	200186	NM_181715	GCUCAGGGCCUAACAUCAU
L-018947-00	J-018947-08	CRTC2	200186	NM_181715	CAGGCAGGCAGUCUCAUUA
L-009678-01	J-009678-09	MPP6	51678	NM_016447	GGAUGAAAGUGCACGGAUU
L-009678-01	J-009678-10	MPP6	51678	NM_016447	GGGAAUGAUAGAUCGACAA
L-009678-01	J-009678-11	MPP6	51678	NM_016447	CUACCAAGCUUCUGACCGA
L-009678-01	J-009678-12	MPP6	51678	NM_016447	CAGUAGAUGCCAUUCGUAU
L-017572-00	J-017572-05	WBP2	23558	NM_012478	GCAAUCCUCACAACGUCUA
L-017572-00	J-017572-06	WBP2	23558	NM_012478	GCGGAGUGAUCGUCAAUAA
L-017572-00	J-017572-07	WBP2	23558	NM_012478	CAUGAAAGACUGUGAGAUC
L-017572-00	J-017572-08	WBP2	23558	NM_012478	GCCUAUGGCUACUCUUACA
L-017900-01	J-017900-09	KCMF1	56888	NM_020122	AAAAUGGGCUAUACGGAGA
L-017900-01	J-017900-10	KCMF1	56888	NM_020122	UCCAAUAUGUGCAGCGUUA
L-017900-01	J-017900-11	KCMF1	56888	NM_020122	UGUCUGAAACGGAGCGCCA
L-017900-01	J-017900-12	KCMF1	56888	NM_020122	GAAAGUGGUGCAACAACAA
L-012200-00	J-012200-05	YAP1	10413	NM_006106	GCACCUAUCACUCUCGAGA
L-012200-00	J-012200-06	YAP1	10413	NM_006106	UGAGAACAAUGACGACCAA
L-012200-00	J-012200-07	YAP1	10413	NM_006106	GGUCAGAGAUACUUCUUAA
L-012200-00	J-012200-08	YAP1	10413	NM_006106	CCACCAAGCUAGAUAAAGA
L-004068-00	J-004068-09	DVL1	1855	NM_181870	GACCAAGGCCUAUACAGUG
L-004068-00	J-004068-10	DVL1	1855	NM_181870	GCGAGUUCUUCGUGGACAU
L-004068-00	J-004068-11	DVL1	1855	NM_181870	GAUCACACGGCACCGAGUG
L-004068-00	J-004068-12	DVL1	1855	NM_181870	GGGGAUCUCUGCAGCAAUC
L-021531-02	J-021531-11	SRGAP2	23380	NM_001042758	CCACACGAGCGAUGACGAA
L-021531-02	J-021531-13	SRGAP2	23380	NM_001042758	GCACAAUGGCAUCGACGGA
L-021531-02	J-021531-22	SRGAP2	23380	NM_001042758	CUGCUUUACCAGCGGGCUU
L-021531-02	J-021531-23	SRGAP2	23380	NM_001042758	GGGUCAUGUAGCCGAUAUU
L-018808-01	J-018808-09	ENY2	56943	NM_020189	GGCACACUGUAAAGAGGUA
L-018808-01	J-018808-10	ENY2	56943	NM_020189	GAGCAGCGAUUAACCAAAA
L-018808-01	J-018808-11	ENY2	56943	NM_020189	AGAGAACGCCUCAAAGAGU

L-018808-01	J-018808-12	ENY2	56943	NM_020189	GCUGGAAGGAUCAGUUGAA
L-030275-01	J-030275-09	NYNRIN	57523	NM_025081	CCUACAAGAAGUUGCGUUU
L-030275-01	J-030275-10	NYNRIN	57523	NM_025081	AGACAAAGUUAGCUCGGAU
L-030275-01	J-030275-11	NYNRIN	57523	NM_025081	CUGGAGAGUCUGCCGAAUA
L-030275-01	J-030275-12	NYNRIN	57523	NM_025081	GGGUCAAGCUGAACGCCUU
L-008621-01	J-008621-09	TRAPPC10	7109	NM_003274	GGUUAAUAGUGAUAGUUGA
L-008621-01	J-008621-10	TRAPPC10	7109	NM_003274	GCUGUGAUACCGAAGUGUA
L-008621-01	J-008621-11	TRAPPC10	7109	NM_003274	CGAAGAUGAUUCACCUAGA
L-008621-01	J-008621-12	TRAPPC10	7109	NM_003274	UCUCUCAGUAUGUGGUCAA
L-012603-00	J-012603-05	TEAD1	7003	NM_021961.5	CGAUUUGUAUACCGAAUAA
L-012603-00	J-012603-06	TEAD1	7003	NM_021961.5	CACAAGACGUCAAGCCUUU
L-012603-00	J-012603-07	TEAD1	7003	NM_021961.5	AAACAGGGAUACACAAGAA
L-012603-00	J-012603-08	TEAD1	7003	NM_021961.5	GAAAGGUGGCUUAAAGGAA
L-012604-00	J-012604-05	TEAD3	7005	NM_003214.3	GGAAGAAGGUGCGGGAGUA
L-012604-00	J-012604-06	TEAD3	7005	NM_003214.3	CGCCGACGCUCAGCAGUUA
L-012604-00	J-012604-07	TEAD3	7005	NM_003214.3	UUGAUUGCACGCUAUAUUA
L-012604-00	J-012604-08	TEAD3	7005	NM_003214.3	GAUCGUCUCUGCCAGUGUC
L-019570-00	J-019570-08	TEAD4	7004	NM_003213.3	GACAGAGUAUGCUCGCUAU
L-019570-00	J-019570-09	TEAD4	7004	NM_003213.3	GGACACUACUCUUACCGCA
L-019570-00	J-019570-10	TEAD4	7004	NM_003213.3	UCAAGCACCUCCCUGAGAA
L-019570-00	J-019570-11	TEAD4	7004	NM_003213.3	CCCAUGAUGUGAAGCCUUU

Supplementary Methods

Genome-wide pooled shRNA screens

Library preparation, cell infection, and screening DECIPHER lentiviral pooled shRNA libraries were obtained from Cellecta **(http://www.cellecta.com/products-services/cellecta-pooled-lentiviral-libraries/decipher-shrna-libraries/).** They consisted of three separate modules (human modules 1, 2 and 3) with each module having 27,500 sequences and covering approximately 5,000 genes, each gene being targeted by 5–6 shRNAs (see Supplementary Data 1 for list of targeted genes). In total, the pooled shRNA library targeted 15,256 REFSEQ genes. Each shRNA construct in the pools contains a unique 18nt barcode which allows for identification by next generation sequencing. Lentiviral particles were generated in HEK-293T cells by co-transfection of the pooled shRNA library with the packaging plasmids psPAX2 and pMD2.G (obtained from Addgene), as previously described[1]. Viral particles were concentrated using Lenti-X (Clontech) and viral titre was determined by flow cytometry using the RFP signal generated by the lentiviral constructs. For each screen the three different shRNA modules (modules 1, 2 and 3) were treated as separate shRNA pools and data was only merged after next generation sequencing and barcode deconvolution. For the screens two independent experiments were performed using either NHKs (strain km, passage 2) or SCC13 cells. NHKs and SCC13 cells were cultured in complete FAD medium on a feeder layer of 3T3-J2 fibroblasts, pre-treated with 4 µg ml^{-1} mitomycin-C (Sigma-Aldrich)[2, 3]. 1.2 x 10^8 NHKs or SCC13 cells were infected at a MOI of 0.5 (so that 50% of cells carried a single shRNA construct) for each shRNA module (1, 2 and 3). Therefore, each individual shRNA was represented by ~2,000 cells. 24h after infection, half of the infected cell population (6 x 10^7 cells per module) was collected for genomic DNA purification (t=0). The other half was seeded at clonal density (6 x 10^5 cells per 175 cm^2 feeder layer) into 10 layer 175cm^2 Corning® HYPERflasks® (Sigma-Aldrich) and cells were cultured for two weeks (t=14).

Sample-preparation, PCR pre-amplification, and next generation sequencing Genomic DNA (gDNA) was isolated from NHK and SCC13 cells by phenol/chloroform purification. 400 µg of gDNA per replicate was used as template in a pre-amplification PCR reaction with Phusion® High Fidelity Polymerase (NEB) to amplify an 180bp amplicon containing the 18nt barcode sequence, which is unique to each shRNA construct. The 180bp PCR products

containing Illumina sequencing adapters were run on a 2% (w/v) agarose gel, and a clean band was excised and purified using QIAquick Gel Extraction kit as recommended by the manufacturer (Qiagen). Samples were then sequenced using Illumina HiSeq (50bp single end-reads).

Sequence processing, quantification of relative shRNA abundance, and identification of gene target hits Illumina sequencing reads were trimmed to the 18nt barcode sequence using the galaxy genome browser (**https://usegalaxy.org/**) and shRNAs were matched to their 18nt barcode using the Barcode Deconvolution Software (Cellecta), with a maximum barcode mismatch of 1bp. Illumina sequencing reads were of high quality (Supplementary Fig. 2), and they mapped to the shRNA library with predictable efficiency and indicated almost complete coverage of the pool (Supplementary Fig. 3a, b). Differential representation of each shRNA in the three modules was determined using DESeq2[4], which is an R package, part of the Bioconductor framework[5]. DESeq2 uses a model based on the negative binomial distribution to estimate the significance of fold change and to account for biological variability among biological replicates. It estimates the dispersions (variability) for each shRNA in the pool and applies the Benjamini-Hochberg multiple test correction to the reported p values. The DESeq2 data from each shRNA module was merged into an output file containing for each shRNA the gene annotation, the total and averaged Illumina sequencing reads from each individual experiment (N1, N2) at t=0 and t=14, the average fold change in abundance when comparing t=14 vs t=0, and the Benjamini-Hochberg multiple test corrected p value (Supplementary Data 3). To identify and rank the gene target hits, we developed a bioinformatics pipeline (Supplementary Fig.1, Supplementary Data 2). We first compared the representation of all shRNAs at t=0 and t=14. shRNAs with <100 reads at t=0 or <20 reads at t=14 were considered below background noise and were excluded. Less than 0.5% of associated candidate gene targets did not pass this filter (Supplementary Data 2). The absence of these shRNAs could be because of defectiveness of the original viral library or early cellular toxicity. We next filtered for statistical significance and considered only shRNAs with a p-value <0.05 for further downstream analysis (Supplementary Fig.1, Supplementary Data 2). All negative control shRNAs in the pool (targeting luciferase) were filtered out at this step. Following this filter we introduced the "messy factor" to score for each gene target whether all respective shRNAs in the set (displaying a statistically significant difference in abundance (p <0.05)) followed the same trend. Only gene target hits with a messy factor of 0 were considered for further analysis. This meant that all statistically significant shRNAs in a set targeting a given gene had to be

either over- or underrepresented at t=14, and no shRNA in the set was allowed to exhibit a change in the opposite direction (Supplementary Fig.1, Supplementary Data 2). Gene target hits were then ranked according to whether there was an average log_2-fold change greater than 0.7 of normalized shRNA read abundance for at least two shRNAs targeting the given gene (Fig. 1f,g; Supplementary Fig. 3c,d; Supplementary Data 2,3). shRNAs targeting non-essential growth regulators were expected to be maintained at a neutral abundance due to a balance between growth of infected stem cells and loss of infected committed progenitors by differentiation and detachment. All in-house computational code is available upon request.

Gene ontology analysis, GSEA pathway analysis and Ingenuity analysis Gene target hit lists were split into candidate positive and negative growth regulators, depending on whether the associated average log_2-fold change in shRNA abundance was negative or positive, respectively. Each sub-list was then imported into the GSEA MSig DataBase[6, 7] and we computed the gene overlap with the gene sets in C2:Canonical pathways gene sets and in C5:GO gene sets BP (GO biological process) and MF (GO molecular function) (Supplementary Data 4, 5). The sub-list of SCC13 candidate positive growth regulators was imported into Panther[8] (Supplementary Data 5). The ranked gene target hit lists were also imported into IPA Ingenuity Pathway analysis and a core analysis was performed (Supplementary Data 4,5).

AP1-signalling factors We mined our candidate growth regulators for AP-1 signalling factors by comparing our ranked gene target hit lists to a list of AP-1 signalling factors compiled from PathCards (**http://pathcards.genecards.org/**) (Supplementary Data 6).

WNT signalling pathway We mined our candidate growth regulators for the WNT signalling pathway components by comparing our ranked gene target hit lists to a list of WNT signalling pathway components obtained from the TaqMan® Array Human WNT Pathway (ThermoFisher; Supplementary Data 6).

Collection and processing of cutaneous SCC (cSCC) gene expression data and compilation of a cSCC gene expression signature We pooled 5 data sets comprising microarray data from clinically annotated cSCC tissue and cell samples (see table below) to compile a list of genes associated with cSCC. This list was then matched against our ranked gene target hit lists for NHKs and SCC13 cells (Supplementary Data 7).

Study	cSCC samples	Normal tissue samples
Dooley et al, 2003[9]	6 cell lines + 4 biopsies	1 biopsy
Mitsui et al, 2014[10]	8 biopsies	10 biopsies
Ra et al, 2011[11]	6 biopsies	5 biopsies
Kathpalia et al, 2006[12]	5 biopsies	5 biopsies
Haider et al, 2006[13]	8 biopsies	8 biopsies

YAP/TAZ/TEAD target gene signature By combining two available YAP/TAZ/TEAD ChIP-seq datasets[14, 15] we were able to compile a compendium of 6197 YAP/TAZ/TEAD gene targets (Supplementary Data 8). This list was then overlapped with our ranked gene target hit lists to identify the YAP/TAZ/TEAD gene targets among our candidate growth regulators of NHKs and SCC13 cells.

Hippo network and YAP interactomes We pooled three published proteomic interaction data sets[16-18] to create a comprehensive interactome list for the human Hippo signalling network. We then added to this list the data available from IPA that is annotated for direct interactions with a confidence level of 'experimental observed'. Subsequently, we mapped our ranked gene target hit lists to the Hippo network interactome (Supplementary Data 9,10). The Hippo Pathway representation in Fig. 1i, j was obtained from IPA software and each node can either represent a single gene/protein or a gene/protein family. The ranked gene target hit lists for NHKs and SCC13 cells were respectively overlaid on the pathway diagram.

Human epidermal stem cell- and terminally differentiated cell-specific gene regulatory regions Our ranked gene target hit lists were mined against the list of genes associated with human epidermal stem cell-specific regulatory regions identified by Cavazza et al.[19] (Supplementary Data S11), and the list of genes associated with human epidermal stem cell- and terminally differentiating cell-specific enhancers identified by Rinaldi et al.[20] (Supplementary Data S12) .

Supplementary references

1. Benaich, N. *et al.* Rewiring of an epithelial differentiation factor, miR-203, to inhibit human squamous cell carcinoma metastasis. *Cell reports* **9**, 104-117 (2014).
2. Jones, P.H. & Watt, F.M. Separation of human epidermal stem cells from transit amplifying cells on the basis of differences in integrin function and expression. *Cell* **73**, 713-724 (1993).
3. Rheinwald, J.G. & Green, H. Serial cultivation of strains of human epidermal keratinocytes: the formation of keratinizing colonies from single cells. *Cell* **6**, 331-343 (1975).
4. Anders, S. & Huber, W. Differential expression analysis for sequence count data. *Genome biology* **11**, R106 (2010).
5. Gentleman, R.C. *et al.* Bioconductor: open software development for computational biology and bioinformatics. *Genome biology* **5**, R80 (2004).
6. Liberzon, A. *et al.* Molecular signatures database (MSigDB) 3.0. *Bioinformatics* **27**, 1739-1740 (2011).
7. Subramanian, A. *et al.* Gene set enrichment analysis: a knowledge-based approach for interpreting genome-wide expression profiles. *Proceedings of the National Academy of Sciences of the United States of America* **102**, 15545-15550 (2005).
8. Mi, H., Muruganujan, A. & Thomas, P.D. PANTHER in 2013: modeling the evolution of gene function, and other gene attributes, in the context of phylogenetic trees. *Nucleic acids research* **41**, D377-386 (2013).
9. Dooley, T.P., Reddy, S.P., Wilborn, T.W. & Davis, R.L. Biomarkers of human cutaneous squamous cell carcinoma from tissues and cell lines identified by DNA microarrays and qRT-PCR. *Biochemical and biophysical research communications* **306**, 1026-1036 (2003).
10. Mitsui, H. *et al.* Gene expression profiling of the leading edge of cutaneous squamous cell carcinoma: IL-24-driven MMP-7. *The Journal of investigative dermatology* **134**, 1418-1427 (2014).
11. Ra, S.H., Li, X. & Binder, S. Molecular discrimination of cutaneous squamous cell carcinoma from actinic keratosis and normal skin. *Mod Pathol* **24**, 963-973 (2011).
12. Kathpalia, V.P. *et al.* Genome-wide transcriptional profiling in human squamous cell carcinoma of the skin identifies unique tumor-associated signatures. *The Journal of dermatology* **33**, 309-318 (2006).
13. Haider, A.S. *et al.* Genomic analysis defines a cancer-specific gene expression signature for human squamous cell carcinoma and distinguishes malignant hyperproliferation from benign hyperplasia. *The Journal of investigative dermatology* **126**, 869-881 (2006).
14. Stein, C. *et al.* YAP1 Exerts Its Transcriptional Control via TEAD-Mediated Activation of Enhancers. *PLoS genetics* **11**, e1005465 (2015).
15. Zanconato, F. *et al.* Genome-wide association between YAP/TAZ/TEAD and AP-1 at enhancers drives oncogenic growth. *Nature cell biology* **17**, 1218-1227 (2015).
16. Hauri, S. *et al.* Interaction proteome of human Hippo signaling: modular control of the co-activator YAP1. *Molecular systems biology* **9**, 713 (2013).

17. Couzens, A.L. *et al.* Protein interaction network of the mammalian Hippo pathway reveals mechanisms of kinase-phosphatase interactions. *Science signaling* **6**, rs15 (2013).
18. Wang, W. *et al.* Defining the protein-protein interaction network of the human hippo pathway. *Molecular & cellular proteomics : MCP* **13**, 119-131 (2014).
19. Cavazza, A. *et al.* Dynamic Transcriptional and Epigenetic Regulation of Human Epidermal Keratinocyte Differentiation. *Stem cell reports* **6**, 618-632 (2016).
20. Rinaldi, L. *et al.* Dnmt3a and Dnmt3b Associate with Enhancers to Regulate Human Epidermal Stem Cell Homeostasis. *Cell stem cell* **19**, 491-501 (2016).
